# Timing disownership experiences in the rubber hand illusion

**DOI:** 10.1186/s41235-016-0041-4

**Published:** 2017-01-30

**Authors:** Timothy Lane, Su-Ling Yeh, Philip Tseng, An-Yi Chang

**Affiliations:** 1 Graduate Institute of Humanities in Medicine, Taipei Medical University, Taipei, Taiwan; 2 TMU—Research Center for Brain and Consciousness, Taipei Medical University, Taipei, Taiwan; 3 Shuang-Ho Hospital, Taipei Medical University, New Taipei City, Taiwan; 4 Institute of European and American Studies, Academia Sinica, Taipei, Taiwan; 5 Research Center for Mind, Brain, and Learning, National Chengchi University, Taipei, Taiwan; 6 College of Humanities and Social Sciences, Taipei Medical University, Hsin-Yi District, 250 Wu-Hsing Street, Taipei, 11031 Taiwan; 7 Graduate Institute of Health and Biotechnology Law, Taipei Medical University, Taipei, Taiwan; 8 Department of Psychology, National Taiwan University, No. 1, Sec. 4, Roosevelt Road, Taipei, 10617 Taiwan; 9 Graduate Institute of Brain and Mind Sciences, National Taiwan University, Taipei, Taiwan; 10 Neurobiology and Cognitive Neuroscience Center, National Taiwan University, Taipei, Taiwan

**Keywords:** Rubber hand illusion, Disownership experience, Multisensory integration, Onset time, Illusion duration

## Abstract

Some investigators of the rubber hand illusion (RHI) have suggested that when standard RHI induction procedures are employed, if the rubber hand is experienced by participants as owned, their corresponding biological hands are experienced as disowned. Others have demurred: drawing upon a variety of experimental data and conceptual considerations, they infer that experience of the RHI might include the experience of a supernumerary limb, but that experienced disownership of biological hands does not occur. Indeed, some investigators even categorically deny that any experimental paradigm has been employed or any evidence can be adduced to support the claim that disownership experiences occur during the RHI. It goes without saying that RHI experiences can be elusive, and that there is some evidence to support claims that supernumerary limb experiences can sometimes occur. Here, however, we test the claim that the conscious experience of disownership can occur during the RHI. In order to test this claim, we developed two new online proxies—onset time for the illusion and illusion duration—and combined these with established questionnaires that concern the conscious contents of the RHI, in particular ownership/disownership experiences. Both online proxy data and post hoc questionnaire data converge in supporting the claim that disownership experiences do occur, at least when the left hand is the object of investigation. Our findings that onset time and illusion duration are reliable measures suggest that investigations of the RHI stand to benefit by devoting more attention to data collected while the RHI is being experienced, in particular data concerning temporal dynamics.

## Significance

Clinicians occasionally encounter patients who feel that one of their limbs—usually the left arm or leg—belongs to someone other than self. Somatoparaphrenia and body integrity identity disorder are examples of such disorders. This phenomenon—the conscious experience of body disownership—is not well understood. Our research demonstrates how, using an established paradigm, this phenomenon can be induced and investigated in healthy participants.

## Background

### Rubber hand illusion

In daily life we assume that hands belong to us when they are connected to our bodies in the usual way, and that artificial hands not so connected cannot be ours. But Tastevin (1937) produced evidence to suggest that artificial hands could be experienced as belonging to self when participants simply look at an artificial hand that is appropriately aligned with body position and posture (cf., Schaefer, Heinze, & Rotte, 2009; Ferri, Chiarelli, Merla, Gallese, & Costantini, 2013). This belonging or ownership illusion was replicated by Botvinick and Cohen (1998) under experimental conditions that incorporated tactile sensation. The phenomenon, what has come to be known as the rubber hand illusion (RHI), has since been further replicated, by various techniques, multiple times (for reviews, see Ehrsson, 2009, 2012; Makin, Holmes, & Ehrsson, 2008; Tsakiris et al, 2010; Tsakiris 2011), even in non-humans (Wada, Takano, Ora, Ide, & Kansaku, 2016).

In the standard version of RHI, an artificial hand is experienced as belonging to self when a participant observes strokes applied with a paint brush to a rubber hand while, in synchrony, strokes are also being applied to the occluded, biological hand. When strokes are applied asynchronously, the illusion either fails to occur or is less vivid, although individual sensitivity to perceiving asynchrony varies as a function of a temporal binding window (Costantini et al., 2016). Although the RHI often exhibits an elusive quality (e.g., Hohwy, 2013), the subjective experience of ownership can be investigated by means of questionnaires that generate data amenable to psychometric analyses (e.g., Longo, Schuur, Kammers, Tsakiris, & Haggard, 2008). Indeed, because the illusion can develop so quickly, amazed participants often spontaneously report that the rubber hand “comes alive,” that it belongs to self (Ehrsson, 2012). This startling experience of ownership for a “hand” can even be elicited when participants are looking at nothing but empty space, provided that certain minimal conditions are satisfied, in particular the synchrony of stroking (Guterstam, Gentile, & Ehrsson, 2013).

### Is there also a disownership experience?

Although most investigations of the RHI focus on the ownership experience, recently some tantalizing evidence has been adduced to suggest that participants also experience alienation from, or disownership for, the biological hand. For example, Longo et al. (2008; cf., Preston, 2013) discovered that participants deny that they experience having three hands, while they do feel that the biological hand disappears. In a follow-up study that drew upon the same data set (Longo, Schuur, Kammers, Tsakiris, & Haggard, 2009), they adduced further evidence suggesting that disownership can be a significant component of the illusion. Indeed qualitative analyses of the RHI that employed interpretive phenomenological analysis also suggest that participants experience disownership for the biological hand (Lewis & Lloyd, 2010; Moguillansky, O’Regan, & Petitmengen, 2013). A possible mechanism for inducing disownership is the attenuation of neuronal responses in select multisensory regions: that is, these attenuated responses prevent the integration of multisensory signals for the biological hand (Gentile, Guterstam, Brozzoli, & Ehrsson, 2013).

Additional support for the claim that disownership experiences occur during the RHI derives from investigations into homeostatic, sensory, and immunological processes. Moseley et al. (2008), for example, have shown that the illusion engages homeostatic processes in such a way that the skin temperature of the biological hand decreases when participants experience ownership for the rubber hand, apparently because experienced disownership causes a selective reduction in blood flow (see also Hohwy & Paton, 2010; Kammers, Rose, & Haggard, 2011; Moseley, Gallace, & Spence, 2012). These findings, however, should be taken with a grain of salt for, in personal communication, J. Hohwy and others have noted that this experiment is not replicated easily; indeed, our group also failed to detect significant decreases in skin temperature.^1^ Most importantly, one study by Rohde, Wold, Karnath, and Ernst (2013) demonstrated that the cooling effect may be present in both the synchronous and the control condition, suggesting that the temperature effect may not be a direct result of the illusion.

In a separate experiment concerning sensory processing, Moseley et al. (2008) have also shown that RHI vividness correlates with a diminished ability to accurately determine the sequence in which tactile stimuli are delivered to the index fingers of the left and right biological hands. They attribute this diminished ability to a decrease in weighting given to tactile information from the biological hand. And Barnsley et al. (2011) have shown that the vividness of the illusion correlates with elevated histamine activity in the biological hand. This elevation of activity they interpret as suggesting that the biological limb is being “rejected.” These findings converge in lending support to the view that experienced disownership might be an important component of the RHI.

But the claims about disownership have not gone unchallenged; investigations of limb disownership have not produced conclusive results (Guterstam & Ehrsson, 2012). Schutz-Bosbach, Tausche, and Weiss (2009), for example, designed an experiment aimed at determining whether or how conceptual interpretations of visual and tactile sensations influence the RHI. Their findings led them to infer that the biological hand is retained, not disowned. Folegatti, de Vignemont, Pavani, Rossetti, and Farne (2009) reached similar conclusions. They employed prismatic lenses to create a visual-proprioceptive conflict, albeit one that does not engender the experience of ownership for the rubber hand. They then compared results from this test to results from a standard RHI that includes ownership. Because in both cases they detected somatosensory changes in the biological hand (viz., a slowing of reaction time to tactile stimuli), they suggest that these objective changes result not from disownership of the hand, but from the brain not “knowing” where the biological hand is. Finally, they venture the categorical claim that “there is no experimental setup that can artificially induce the explicit sensation of disownership of one’s own hand.”

de Vignemont (2011) has also challenged the claim that disownership experiences occur; indeed, she too categorically asserts that there is “no evidence” to support such claims. Her concerns are a mix of the conceptual and the experimental. But she has clearly articulated several issues that contribute to confusion over what has been learned from RHI experiments, and what can be learned. She agrees with the commonplace observation that reports of the experience are variable and elusive, and then she underscores the possible role that a widespread failure to distinguish between the “experience of” and the “judgment of” ownership might play in making this illusion so difficult to nail down. She also points out that experimental paradigms tend to rely excessively on questionnaires, tend to pay inadequate attention to updating of “online representations of bodily properties”, and too often neglect observed dissociation between what one experiences and proprioceptive drift, the most frequently employed objective measure of ownership and disownership.

### Aims of this study

Within the framework of the RHI paradigm, there is ample evidence that an illusion of ownership occurs for the rubber hand. What remains unclear is whether, within that same framework, an illusion—a conscious experience—of disownership can occur for the biological hand. Moreover, if there is in fact such an experience, it remains unclear how stable or how robust it might be.

Our experiments focus on the contents of conscious experience within the RHI, devoting special attention to disownership. For this reason we designed a series of experiments that began by first distinguishing clearly between what is experienced and what is inferred or judged to be the case, while emphasizing that our concern is with the former. Second, given the importance of subjective report to our experimental aims, we drew upon multiple, previously employed questionnaires, seeking to determine whether responses to similar content that was differently phrased would converge. Third, given the seeming elusiveness of the RHI as well as the lack of adequate information concerning how it is experienced online, we employed novel, online proxies—onset time (OT) and illusion duration (ID). Finally, because some evidence suggests that laterality is a factor relevant to the emergence of disownership experiences, we concentrated on the left rather than on the right hand.

Alterations of the standard RHI paradigm suggest that ownership experiences can be retained for the biological hand (Ehrsson, 2009; Schaefer et al., 2009; Newport, Pearce, & Preston, 2010; Guterstam, Petkova, & Ehrsson, 2011). Our aim is not to challenge these findings. Instead, our aim is to test the categorical denials that disownership experiences can occur within the RHI, by collecting data on conscious experience as it is reported, both while the experience is ongoing and after the fact.

### Distinctive methodological approach

A commonly used behavioral proxy for the RHI is proprioceptive drift. But several studies have raised doubts about the reliability of drift as an indicator of the ownership experience for the rubber hand (Rohde, Luca, & Earnst, 2011; Zopf, Savage, & Williams, 2010; Ehrsson, Spence, & Passingham, 2004; Fiorio et al., 2011; Kammers et al., 2009). A more recent study, however, seems to suggest that although drift and ownership experience are positively correlated, crucially, it is the conscious experience that seems to be causing drift, not the reverse (Abdulkarim & Ehrsson, 2016). This is one of the factors motivating our focus on the conscious contents of the RHI.

In view of the seeming unreliability of proprioceptive drift, our focus on conscious experience, and the evidence suggesting that conscious experience causes drift, we opted not to employ this behavioral proxy. Instead, we used two online temporal measures: OT and ID. A few prior studies have recorded OT (Ehrsson et al., 2004; Ehrsson, Wiech, Weiskopf, Dolan, & Passingham, 2007; Lloyd, 2007), but previously the temporal dimension—whether OT or ID—has never been employed as a proxy for the RHI. Peled, Ritsner, Hirschmann, Geva, and Modai (2000), despite not using OT as a proxy, did notice that OT and illusion strength correlate in patients with schizophrenia. We extrapolated from this incidental finding, as well as the observation that the illusion can begin as early as 10–20 s after the start of synchronous stimulation (Lloyd, 2007; Ehrsson et al., 2004), and designed three experiments that explicitly treated the temporal dimension, both OT and ID, as potential online proxies for healthy participants; OT was used for the first two experiments, and ID was used for the third. Although OT might seem problematic, given that in some cases the illusion can begin at the very moment the participant observes the rubber hand, previous data nevertheless suggest that waiting time tends to range between 5 and 116 s, with 27 s being the median (Slater, Perez-Marcos, Ehrsson, & Sanchez-Vives, 2009).

The first experiment aimed at ascertaining whether our claim that OT and illusion strength correlate in healthy subjects could be confirmed, even without first suggesting to the participants what type of conscious content was under investigation. The second experiment applied findings from the first in order to directly test the disownership claim by combining a psychometric approach to post hoc questionnaire data with online OT data. Finally, to further confirm findings from the second experiment, the third experiment employed a different questionnaire, a different scale, and a different online proxy, albeit one that also involves the temporal dimension ID.

Surely even naïve participants could discern from the experimental context that some experience relevant to the rubber hand might be expected. But it is not self-evident that this experience will be of ownership or disownership illusions. As a matter of fact, individual difference in how the RHI is experienced is to be expected (Haans, Kaiser, Bouwhuis, & Ijsselsteijn, 2012). It could just as easily be only touch referral—the feeling of touch on the rubber hand—that is experienced, absent the richer phenomenology involving ownership, because touch referral is actually the illusion’s most distinctive perceptual event (Ehrsson, 2012).

Accordingly, in order to avoid suggesting any specific conscious content, for the first experiment we included a common vertical–horizontal illusion in our instructions, both to illustrate what an illusion is and to distinguish illusion from inference or judgment. Participants were told to indicate OT the moment they experienced an illusion of some kind. We did not divulge to them that the experiment was an investigation of ownership–disownership experiences. Instead, to determine whether participants experienced such illusions, we adopted items from the Longo et al. (2008) questionnaire that had previously been identified as relevant to ownership for the rubber hand and disownership for the biological hand.

Emphasizing the experience–inference distinction is important for this first experiment. A principal reason is that prior results seem to strongly suggest that an ownership experience for the rubber hand can occur. But the contentious point is whether a complementary disownership experience can occur for the biological hand. The worry is that some participants who have an ownership experience might then infer or judge that their biological hand must have been disowned, even if they had no such experience. Avoiding conflation of experience and inference is crucial to clearly determine the conscious contents of the RHI.

Having in the first experiment established the reliability of OT as an online, in-the-moment proxy for the RHI in healthy participants, and having established that participants reported ownership and disownership experiences without either having been suggested to them prior to the experiment, in the second experiment we focused even more directly on the experience of disownership. Again we used the Longo et al. (2008) questionnaire items in conjunction with OT measurements. But here participants were instructed to use OT to indicate the moment of onset of either disownership or ownership. For the third experiment, we sought additional confirmation of findings concerning use of a temporal, online proxy, by using ID, and additional confirmation of subjective report by using an alternative questionnaire and scale (Preston, 2013).

Throughout we focused only on the left hand. The principal reason is that right hemisphere tactile activation tends to evince a more vivid illusion of ownership for the rubber hand (Ocklenburg, Ruther, Peterburs, Pinnow, & Gunturkun, 2011). On the assumption that experiences of ownership and disownership are intrinsically related, we conjectured that right hemisphere tactile activation also tends to evince a more vivid illusion of disownership. In fact, certain pathologies suggest that disownership experiences are lateralized in just this way (Vallar & Ronchi, 2009; Karnath & Baier, 2010; Giummarra, Bradshaw, Hilti, Nicholls, & Brugger, 2012).

### Significance of this study

Schutz-Bosbach et al. (2009), Folegatti et al. (2009), de Vignemont (2011), and others adduce evidence to infer that standard RHI induction procedures do not or cannot induce disownership experiences for one’s biological hand. Our investigations are designed to test their views (1) by focusing on the conscious content of the RHI; (2) by emphasizing the difference between experience and inference or judgment; (3) by collecting data online, while participants are having the experiences, and after the fact, using dual measures for both; and (4) by focusing on just one hand, the left. Proceeding in this way we are able to marshal support for the view that not only can disownership occur during the RHI, but also that disownership is consciously experienced.

## Experiment 1

### Overview and hypothesis

Two measures were used to determine whether standard RHI induction procedures bring about disownership experiences. First, the OT was recorded while biological and rubber hands were being stroked. Second, after completion of the experiment, a questionnaire designed to probe ownership of the rubber hand and disownership of the real hand was administered. If standard RHI induction procedures engender disownership, the questionnaire should evince both ownership of the rubber hand and disownership of the biological hand, while the strength of both should positively correlate with OTs. If standard RHI induction procedures do not engender disownership, however, it should neither be evinced by the questionnaire nor correlate with OTs.

### Methods

#### Participants

Thirty-two undergraduate participants were recruited for this experiment. Because no previous studies had used OT as an online proxy for the RHI, in order to confirm that despite some degree of individual difference it could still serve as a valid measure, we increased the sample size for standard, behavioral versions of the RHI experiments from the typical 10–20 to 32. All participants had normal or corrected-to-normal vision and no known tactile deficits. All were naïve about the purpose of this experiment, and no one among them had previously participated in a RHI experiment. This study was approved by the Department of Psychology Ethics Committee, National Taiwan University, and all participants gave informed consent.

#### Experimental setup

Participants were individually tested in a small, quiet room. The experimenter stood in front of the participant, who was seated with both hands placed on a table top. Prior to beginning, participants were shown a vertical–horizontal illusion and were advised that the test was designed to see whether or not they would experience an illusion, of some unspecified kind. The purpose of showing them this illusion was to illustrate the difference between what is experienced and what is judged to be the case. As has been emphasized in the previous section, given our special focus on the conscious contents of the RHI, along with skepticism expressed by some investigators that the experience of disownership even occurs, we found clarification of this distinction to be crucial to our design.

**Fig. 1:**
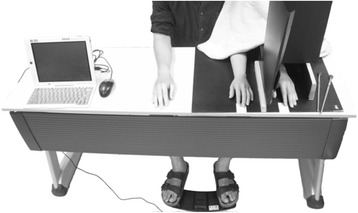
The setup in Experiment 1, 2, and 3

**Fig. 2:**
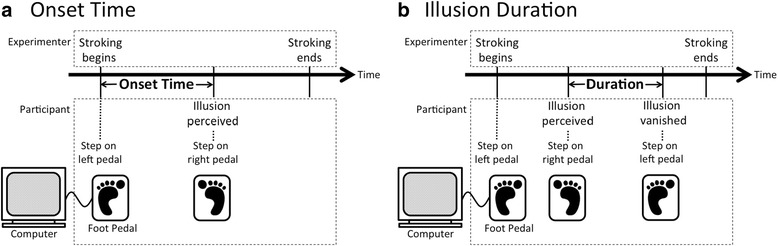
Temporal dimension as a measure of the rubber hand illusion, both for ownership and disownership. **a** Onset time; **b** illusion duration. Concerning duration, if a participant’s illusion comes and goes, illusion duration is the sum of all illusion experience periods

First, participants were asked to insert their hands into a black cardboard tube, so that they would be hidden from view. Second, a towel was placed over the tube and in such a way that it concealed participants’ elbows and forearms, as well as the space separating the rubber hand from the body. Third, the experimenter proceeded to use two paintbrushes to stroke corresponding fingers of the rubber and biological hands, at an approximate rate of one stroke per 2 s, and continuing for a period of 5 min (Fig. 1). Because evidence suggests right hemispheric dominance for the experience of body ownership (Ocklenburg et al., 2011), strokes were applied to the real left hand and a corresponding left, rubber hand. The left hand was used for all three experiments. Two foot pedals connected to a computer were placed under the participants’ feet to measure OTs (Fig. 2a).

#### Design and procedure

Two conditions were administered for each participant: synchronous and asynchronous stroking, with the order counterbalanced across participants. In both conditions the experimenter stroked the rubber hand and participants’ real hands, attempting to induce the RHI. Participants were required to keep looking at the rubber hand and to avoid postural adjustments. As soon as the experimenter started stroking both the rubber and biological hands, participants were required to step on the left pedal. Participants had also been instructed: “During the course of the experiment, if you experience an illusion, please step down on the right pedal, immediately.” After stroking of the hands was completed, participants were asked to fill out a questionnaire (Appendix). Finally, upon completion of the questionnaire, the entire procedure was repeated for the other condition: that is, if the first condition involved synchronous stroking, the second would be asynchronous.

#### Questionnaire

The questionnaire comprised ten items adopted from Longo et al. (2008). To focus on the subjective report of experiencing ownership for the rubber hand and disownership for the real hand, we selected the ownership and disownership (or, “loss of own hand”) items from the study of Longo et al. Items 1–5 reflected rubber hand ownership; items 6–10 reflected real hand disownership. Responses for each item were indicated on a seven-point Likert scale, from −3 to 3. Positive responses to item 7, “It seemed like I could have moved my hand if I had wanted”, were coded inversely (viz., a response of +3 was coded as −3).

Because of the concern that investigations of this sort fail to adequately distinguish between what is experienced and what is judged to be the case (e.g., de Vignemont, 2011), before responding to the questionnaire participants were instructed to base their ratings on what they consciously experience rather than on what they judged or inferred to be the case. As noted, a common vertical–horizontal illusion was used to illustrate the distinction before participants took part in the experiment. It was emphasized to participants that the phenomenon of interest was analogous to experiencing two lines of equal length as though they were of different length, in the sense that what matters is “how things seem to be”. In this way, we tried to minimize the possibility that participants who experienced ownership then inferred that disownership must have also occurred.

## Results for Experiment 1

### Onset time for the illusion

**Fig. 3:**
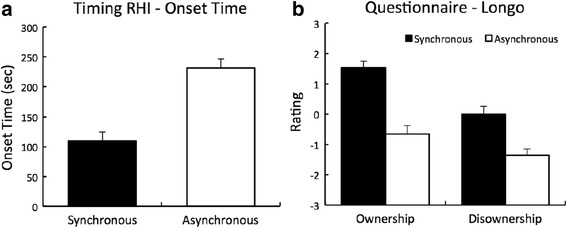
Experiment 1 results. **a** Mean onset times measured under synchronous and asynchronous stroking conditions; **b** mean rating scores for ownership and disownership items. *RHI* rubber hand illusion

We used mean ± 2.5 standard deviations (SD) as cut-off criteria and no participants had to be excluded in follow-up analyses. By measuring the elapsed time from the moment that the left foot pedal was pressed (when stroking of hands begins) to the moment when the right foot pedal was pressed (when participants begin experiencing the RHI), we obtained the OT of the RHI for each participant. For trials when participants did not step on the right pedal before cessation of the stroking, OT was recorded as 300 s (duration of stroking); this ensured that no cells were left empty. (In Experiment 3 we show that this methodological compromise did not contaminate our data.) A one-way repeated measure analysis of variance (ANOVA) on OT showed a significant difference between synchronous and asynchronous stroking (*F*(1, 31) = 34.193, *p* < 0.001, η_p_^2^ = 0.5245). In other words, participants experienced RHI more quickly when the stroking was synchronous than when it was asynchronous (Fig. 3a).

### Questionnaire

We categorized items 1–5 as ownership items, and 6–10 as disownership items. We then conducted a two-way repeated measure ANOVA concerning the synchronous–asynchronous difference and ownership type—that is, ownership versus disownership items (Fig. 3b). Both the main effects of the synchrony–asynchrony difference (*F*(1, 31) = 59.907, *p* < 0.001, η_p_^2^ = 0.6590) and ownership type (*F*(1, 31) = 48.643, *p* < 0.001, η_p_^2^ = 0.6108) were significant. Moreover, interaction between the synchrony–asynchrony difference and ownership type was also significant (*F*(1, 31) = 6.876, *p* < 0.05, η_p_^2^ = 0.1815). Simple main effects revealed that both ownership and disownership items were rated more positively under the synchronous condition than under the asynchronous condition, and ownership items were rated more positively than disownership items under both synchronous and asynchronous conditions. None of the post hoc Tukey tests were significant.

To test whether there was any relationship between the experiences of rubber hand ownership and biological hand disownership, we calculated the correlation between scores of ownership items and disownership items, using the difference scores between the synchronous and the asynchronous conditions. We discovered a significant correlation between ownership and disownership (*r* = 0.38, *p* < 0.05). That is to say, the more strongly a participant experienced a feeling of ownership for the rubber hand, the more strongly they experienced a feeling of disownership for the real hand.

### Correlation between onset time and questionnaire

To further probe the relationship between strength of the illusion as reflected by questionnaire ratings and the emergence of the illusory experience, we calculated two correlations. Both the correlations between OT and ownership items (*r* = −0.39, *p* < 0.05) and between OT and disownership items (*r* = −0.35, *p* < 0.05), when the asynchronous condition was subtracted from the synchronous condition, were significant. These two correlations revealed that the more quickly participants experienced the illusion, the stronger were the ratings of ownership for the rubber hand and disownership for the biological hand items, and vice versa. In short, the experience of RHI is reflected not only by questionnaire item ratings, but also by OT.

## Discussion for Experiment 1

In this experiment, we established that ownership and disownership experiences were identifiable to naïve participants, even though it was only suggested to them that they would experience an illusion of some kind. Second, we have shown that OT can be a reliable online proxy for measuring these two experiential aspects of the RHI: OT correlated with the vividness of ownership for the rubber hand, as well as vividness of disownership for the biological hand. That is, the earlier the onset of the RHI, the stronger the experience of ownership for the rubber hand and disownership for the biological hand, as reflected by questionnaire ratings. Third, we showed a significant positive correlation between ownership for the rubber hand and disownership for the biological hand. These results support the disownership view; they suggest that during the standard RHI induction procedure, at least when it is applied to the left hand, if the rubber hand is experienced as owned, the biological hand is likewise consciously experienced as disowned. Indeed, the more strongly participants experienced ownership for the rubber hand, the more strongly they experienced disownership for the biological hand.

## Experiment 2

### Overview and hypothesis

In Experiment 1, we asked participants to indicate the moment at which they began to experience an illusion. Questionnaire responses reflected that participants were able to distinguish between the experience of ownership and that of disownership, and that these responses correlated with their online recording of OT. Accordingly, we surmised that the OT measure can also be employed to distinguish between these two experiences when participants are aware of what type of illusion to expect. To verify this, for Experiment 2 we asked participants to indicate the instant when they began to experience ownership of the rubber hand, as well as the instant they began to experience disownership of the biological hand. We predicted that, if the disownership view holds true: (1) OTs for both ownership and disownership would be earlier under the synchronous than the asynchronous condition; (2) ownership and disownership OTs would correlate; and (3) ownership–disownership ratings would correlate with ownership–disownership OTs.

### Methods

#### Participants

Twenty participants were recruited. Because we had already established the validity of timing as an online proxy for the illusion (both ownership and disownership), thereby reducing concerns about individual difference, we adopted the sample size common to prior investigations of the RHI (e.g., Ocklenburg et al., 2011; Preston, 2013; Rohde et al., 2011). All participants were college students, who were naïve as regards the RHI experiment, and all gave informed consent. All had normal or corrected-to-normal vision, and no known tactile deficits. Moreover, all were given a modest remuneration for their participation. This study was approved by the Department of Psychology Ethics Committee, National Taiwan University.

#### Experimental setup, design, procedure, and questionnaire

Methods were the same as those employed in Experiment 1, except for the criteria used to decide when the illusion begins. Here, participants were required to respond based upon two distinct “belonging” criteria (Lane, 2012, 2014, 2015): the experience of ownership for the rubber hand (criterion 1) and the experience of disownership for the biological hand (criterion 2). We asked participants to step on the pedal as soon as they began to feel the rubber hand was their real hand and, likewise, when they began to feel their real hand no longer belonged to them. The order of the criteria was counterbalanced among participants. When participants felt a belonging criterion was satisfied—at the instant of first experiencing ownership or disownership—they were required to step on the pedal, thereby recording the OT. For this experiment, each participant was presented with four conditions: synchronous and asynchronous stroking while testing for disownership, and synchronous and asynchronous stroking while testing for ownership. So as not to confuse participants by repetitive changing of criteria, we balanced conditions of belonging criteria, so that one criterion was conducted with both the synchronous and asynchronous conditions first, before proceeding to the next criterion. In other words, if on the first trial a participant was tested for disownership using synchronous stroking, on the second trial testing would be for disownership using asynchronous stroking, on the third trial testing would be for ownership using synchronous stroking, and on the fourth trial testing would be for ownership using asynchronous stroking.

## Results for Experiment 2

### Onset time for the illusion

**Fig. 4:**
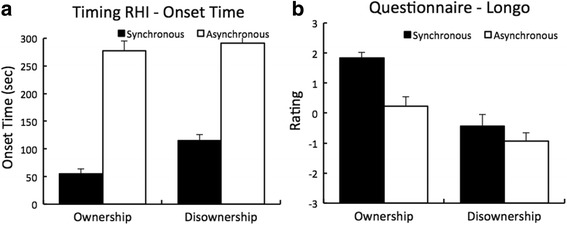
Experiment 2 results. **a** Mean onset times measured under synchronous and asynchronous stroking conditions; **b** mean rating scores for ownership and disownership items. *RHI* rubber hand illusion

We used mean ± 2.5 SD as cut-off criteria and one of the participants was excluded in follow-up analyses. We conducted a two-way repeated measure ANOVA concerning the synchronous–asynchronous difference and belonging type (Fig. 4a). Both the main effects of the synchrony–asynchrony difference (*F*(1, 18) = 339.638, *p* < 0.001, η_p_^2^ = 0.9497) and belonging type (*F*(1, 18) = 32.197, *p* < 0.001, η_p_^2^ = 0.6414) were significant. This suggests that participants experienced the feeling of ownership and disownership more quickly under the synchronous stroking condition than the asynchronous stroking condition, and experienced the feeling of ownership faster than disownership. The interaction between the synchrony–asynchrony difference and belonging type was also significant (*F*(1, 18) = 16.027, *p* < 0.001, η_p_^2^ = 0.4710). The simple main effect reveals that both ownership and disownership were experienced more quickly under the synchronous than the asynchronous condition, and that ownership was experienced more quickly than disownership under the synchronous condition, but that there was no difference between ownership and disownership obtained under the asynchronous condition. These data imply that participants experienced ownership for the rubber hand prior to disownership for the biological hand, but that if they did not experience ownership, neither did they experience disownership.

Furthermore, we assessed the relationship between the OTs of ownership and disownership, when the asynchronous condition was subtracted from the synchronous condition. Here, too, we discovered a significant correlation (*r* = 0.56, *p* < 0.001). This discovery implies that the more quickly participants experience ownership for the rubber hand, the more quickly they experience disownership for the biological hand.

### Questionnaire

We conducted a two-way repeated measure ANOVA concerning the synchronous–asynchronous difference and belonging type (Fig. 4b). Both the main effects of the synchrony–asynchrony difference (*F*(1, 18) = 24.641, *p* < 0.001, η_p_^2^ = 0.5779) and belonging type (*F*(1, 18) = 16.879, *p* < 0.001, η_p_^2^ = 0.4839) were significant. This is to say, participants were more likely to affirm questionnaire items under the synchronous condition than under the asynchronous condition, and ownership items were more strongly affirmed than disownership items. There was also a significant interaction between the synchrony–asynchrony difference and belonging type (*F*(1, 18) = 14.189, *p* < 0.001, η_p_^2^ = 0.4408). In addition, simple main effects revealed that both ownership and disownership items were rated more positively under the synchronous condition than under the asynchronous condition, and ownership items were rated more positively than disownership items under the synchronous condition, but not under the asynchronous condition. None of the post hoc Tukey tests were significant.

In addition, we calculated the correlation between scores of ownership items and disownership items when the asynchronous condition was subtracted from the synchronous condition. We again discovered a significant correlation between ownership and disownership (*r* = 0.69, *p* < 0.001). In short, the stronger the experience of ownership for the rubber hand, the stronger the experience of disownership for the biological hand, and vice versa.

### Correlation between onset time and questionnaire

To further probe the relationship between questionnaire ratings and OTs, we calculated the correlation between ownership questionnaire scores and ownership OTs, as well as the correlation between disownership questionnaire scores and disownership OTs. Both correlations were significant (ownership: *r* = −0.44, *p* < 0.05; disownership: *r* = −0.44, *p* < 0.05). That is, the earlier the onset of ownership or disownership, the more robustly were those illusions experienced.

## Discussion for Experiment 2

In this experiment, having already established that OT can serve as a reliable online proxy for the RHI even when participants are unaware of what type of illusion to expect, we focused even more directly on the disownership hypothesis. Again using the Longo et al. (2008) questionnaire in conjunction with OT measurements, we confirmed that participants could both clearly identify the type of belonging experience and indicate the moment of disownership or ownership onset. Moreover, these OT results significantly correlated with responses to questionnaire items. In sum, Experiment 2 results indicate that OT for both disownership and ownership significantly correlate with questionnaire responses, that disownership and ownership correlate with one another, and that the earlier the onset of either illusion, the more vivid the experience, for both types of belonging experience. The discovery that the more quickly participants experience ownership for the rubber hand, the more quickly they experience disownership for the biological hand supports our conjecture that disownership and ownership are related intrinsically.

## Experiment 3

### Overview and hypothesis

In Experiments 1 and 2, we asked participants to rate ownership items and disownership items adopted from Longo et al. (2008) and to indicate OT. For Experiment 3, three principal changes were made. First, because Longo et al. did not include control items, and in order to determine whether the manner in which ownership–disownership queries were expressed could alter results, we added a questionnaire used by Preston (2013; cf., Guterstam et al., 2011) that includes controls and alternative formulations of questionnaire items. We conjectured that if the ownership–disownership experiences are sufficiently distinct, semantic differences between the two questionnaires would not significantly affect the results. Second, we amended the numerical scale that is typically matched to questionnaires of this sort so that it more clearly indicated whether participants intended to affirm experiencing disownership, as opposed to merely indicating uncertainty. Third, to further explore the utility of the temporal dimension as an online proxy for ownership–disownership experiences, as well as to avoid the need to attribute a 300 s OT to participants who did not experience the RHI, we investigated ID to determine whether it too might evince the same pattern of correlations as those evinced by OT.

### Methods

#### Participants

Twelve participants were recruited. This sample size estimate was based on the large effect sizes obtained in Experiment 2; we therefore reduced the sample size to that which has been adopted for similar RHI experiments (e.g., Botvinick & Cohen, 1998; Folegatti et al., 2009; Morgan et al., 2011). All participants were naïve as regards the RHI experiment, all gave informed consent, and all were given a modest remuneration for their participation. All had normal or corrected-to-normal vision, and no known tactile deficits. This study was approved by the Department of Psychology Ethics Committee, National Taiwan University.

#### Experimental setup, design, procedure, and questionnaire

Stimuli and procedures were very much the same as those employed in Experiments 1 and 2. The critical differences were as follows: first, we gave participants two questionnaires. One was the same as that used previously, while the other (Appendix) contained control items and alternative formulations of questionnaire items (Preston, 2013). The order of the two questionnaires was counterbalanced among participants. Second, rather than using a −3 to +3 Likert scale, we used an 11-point, 0–10 numerical scale. The intent here was disambiguation: for example, on the commonly used Likert scale a selection near 0 might be interpreted as suggesting that the participant experienced nothing. But when the numerical scale only includes positive numbers, a selection near the mid-point more clearly indicates affirmation that an illusion experience occurred.

In order to time the illusion, participants were asked to step down on the right pedal as soon as they experienced the illusion begin and the left foot pedal as soon as the illusion vanished (Fig. 2b). They were also told to repeat this procedure if the illusion started again. The difference between the time points when the two pedals were pressed was calculated as the duration [t(left pedal) – t(right pedal)], and the sum of these durations was indexed as the ID.

## Results for Experiment 3

### Illusion duration

**Fig. 5:**
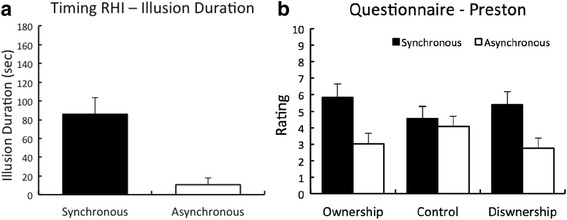
Experiment 3 results. **a** Mean ownership durations measured under synchronous and asynchronous stroking conditions; **b** mean rating scores for ownership, control, and disownership items in Preston’s (2013) questionnaire. *RHI* rubber hand illusion

We used mean ±2.5 SD as the cut-off criteria and no participants had to be excluded from follow-up analyses. We conducted a one-way repeated measure ANOVA concerning the synchronous–asynchronous difference and ID (Fig. 5a). The main effects of the synchrony–asynchrony difference were significant (*F*(1, 11) = 20.409, *p* < 0.001, η_p_^2^ = 0.6498). This shows that participants experienced the feeling of ownership longer under the synchronous stroking condition than under the asynchronous stroking condition.

### Questionnaire

First, we conducted a two-way repeated measure ANOVA concerning the synchronous–asynchronous difference and belonging type based on data collected from Longo et al.’s (2008) questionnaire. The main effects of the synchrony–asynchrony difference (*F*(1, 11) = 9.855, *p* < 0.01, η_p_^2^ = 0.4725) were significant. This is to say, items were rated more positively under the synchronous condition than under the asynchronous condition. There was also a significant interaction between the synchrony–asynchrony difference and belonging type (*F*(1, 11) = 5.210, *p* < 0.05, η_p_^2^ = 0.3214). In addition, simple main effects revealed that both ownership and disownership items were rated more positively under the synchronous than under the asynchronous condition, and ownership items were rated more positively than disownership items under the synchronous condition, but not the asynchronous condition. None of the post hoc Tukey tests were significant.

Second, we conducted a two-way repeated measure ANOVA concerning the synchronous–asynchronous difference and belonging type in Preston’s (2013) questionnaire (Fig. 5b). The main effects of the synchrony–asynchrony difference (*F*(1, 11) = 8.985, *p* < 0.05, η_p_^2^ = 0.4596) were significant. This is to say, items were rated more positively under the synchronous condition than under the asynchronous condition. There was also a significant interaction between synchronous–asynchronous difference and belonging type (*F*(1, 11) = 8.101, *p* < 0.05, η_p_^2^ = 0.4241). In addition, simple main effects revealed that both ownership and disownership items were rated more positively under the synchronous condition than under the asynchronous condition, and ownership items were rated more positively than disownership items under the synchronous condition, but not under the asynchronous condition. As for control items, there was no significant difference between synchronous and asynchronous conditions, indicating that the difference between synchronous and asynchronous in ownership and disownership items was not due to response bias. Moreover, the post hoc Tukey indicated that the ownership items were rated more positively than control items in the synchronous condition (*p* < 0.05) and the disownership items were rated more positively than control items in the synchronous condition (*p* < 0.05). There were also quadratic trends for both the synchronous condition (*F*(1, 44) = 5.940, *p* < 0.05) and asynchronous condition (*F*(1, 44) = 8.093, *p* < 0.01).

Third, we calculated the correlation between scores of ownership items and disownership items when the asynchronous condition was subtracted from the synchronous condition for data collected from both questionnaires. We discovered significant correlations between ownership and disownership in Longo et al.’s (2008) questionnaire (*r* = 0.59, *p* < 0.05) and in Preston’s (2013) questionnaire (r = 0.63, *p* < 0.05). In short, the stronger the experience of ownership for the rubber hand, the stronger the experience of disownership for the biological hand, and vice versa. Moreover, we calculated correlations between the two questionnaires: correlations between ownership items (r = 0.57, *p* < 0.05) and disownership items (r = 0.76, *p* < 0.01) for the two questionnaires were significant.

### Correlation between illusion duration and questionnaire

We further probed the relationship between questionnaire ratings and ID. There were significant correlations between the ownership–disownership questionnaire items of Longo et al. (2008) and ID (ownership: r = 0.65, *p* < 0.05; disownership: r = 0.66, *p* < 0.01), as well as between the ownership–disownership items of Preston (2013) and ID (ownership: r = 0.55, *p* < 0.05; disownership: r = 0.65, *p* < 0.05). In other words, the longer the ID of ownership, the more robustly felt was the illusion.

## Discussion for Experiment 3

Experiment 3 achieved several goals: first, it confirmed the utility of the temporal dimension as an indicator of ownership, while also circumventing the need to make methodological compromises, by demonstrating the ID’s value as an online proxy. Second, results from Experiments 1 and 2 were confirmed using a questionnaire that contained alternative formulations of the questionnaire items as well as control items. Third, the validity of ID as a measure suggests that this illusion is a stable, not transient, phenomenon. In other words, this methodological approach might help to reduce the sense that the RHI is an elusive phenomenon. Finally, by using an 11-point numerical scale, we confirmed that mid-range questionnaire responses are indeed affirmations that an illusion was experienced.

Because one of the Preston (2013) questionnaire control items suggests the possibility of a supernumerary limb, it might be wondered whether data from this item conflicts with our focus on the experience of disownership. Although for future studies of this type it might be advisable to more directly compare supernumerary with disownership items, for our investigation, owing to our special emphasis on subjective report, we decided to use established questionnaires. Furthermore, the disownership items clearly concern disownership, while only one of the control items clearly concerns the possibility of experiencing a supernumerary limb. Finally, the difference between synchrony and asynchrony is more pronounced for the disownership than for the control condition.

## General discussion

### The three experiments

For the first experiment, we emphasized that our concern was with what participants consciously experienced, not what they inferred or judged to be the case; moreover, we avoided suggesting to them how that experience should be characterized. They were told only that they might experience an illusion of some kind, and were instructed to indicate the moment at which that began (OT), if indeed they did experience an illusion. Even without suggesting what might be experienced and despite the caveat to avoid rendering judgments, questionnaire responses and OT combine to reflect that participants did experience both ownership and disownership, and that they experienced both more quickly when hand stroking was synchronous than when it was not.

In addition, questionnaire results showed that the more strongly participants experienced ownership for the rubber hand, the more strongly they experienced disownership for the real hand. Crucially, the more quickly participants indicated the experience of an illusion, the stronger were their questionnaire ratings for both ownership and disownership. Accordingly, it seems that participants do undergo both ownership and disownership experiences and that OT can serve as a useful, online proxy for these experiences, perhaps even as a substitute for drift given that drift seems to be a function of conscious experience.

For the second experiment, having already established that OT can serve as a reliable online proxy in healthy participants, we focused more directly on the categorical denials that disownership experiences occur. This time participants were advised as regards the phenomena under investigation and instructed to indicate OT for both ownership and disownership experiences. Again, ownership and disownership were experienced more quickly when stroking was synchronous than when it was not, and the more quickly ownership was experienced the more quickly disownership was experienced. Furthermore, rapidity of onset for both correlated with the experienced strength of the illusions. Thus, consistent with our conjecture, it seems that for standard versions of the RHI ownership and disownership might be intrinsically related: the degree to which ownership is experienced for the rubber hand, disownership is experienced for the biological hand. When the OT and the questionnaire data were conjoined, they augmented support for the view that disownership experiences do occur.

For the third experiment, to safeguard against the possibility that the Longo et al. (2008) questionnaire items and the rating scale might not adequately disambiguate between disownership and other phenomena, or between disownership and uncertainty, we employed a refined set of ownership–disownership items as well as control items (Preston, 2013), along with a scale better suited to assessing whether participants intended to affirm experience of disownership. Furthermore, in Experiments 1 and 2, methodological concerns compelled us to attribute an OT of 300 s to those who did not experience the RHI. Obviously, this methodological necessity takes certain liberties with the interpretation of “OT”. Accordingly, to demonstrate that attribution of OT to those who did not experience the illusion did not contaminate the data, we employed a different temporal component—ID—to see whether it too could serve as a reliable online proxy. Here, ID was recorded as zero if no illusion was experienced. Not only did we again confirm that the strength of disownership and ownership illusions correlate, we also discovered that ID can, like OT, serve as a useful proxy for these two components of the RHI.

### Disownership and multisensory integration

According to an emerging consensus within cognitive neuroscience, the experience of owning one’s body is dependent upon multisensory integration (e.g., Ehrsson, 2012). Applied to the RHI, the consensus view holds that participants integrate touch felt on the biological hand with visible stroking of the rubber hand, such that they do not experience distinct unimodal events. Instead, they form a coherent representation of a unified, multisensory event—the experience of strokes applied to the rubber hand (Makin et al., 2008). More directly relevant to our concerns here, by virtue of integrating distinct stimuli into a coherent representation, participants then commonly report feeling that the rubber hand belongs to self.

Although it should be noted that this view is not universally shared (e.g., Ferri et al., 2013), our findings are broadly consistent with the multisensory integration consensus. That is, one interpretation of our findings is that disownership experiences result from a dynamic process whereby the body’s representation, within peripersonal space, is regularly updated to account for varied sensory inputs that derive from different modalities. Our findings seem as well to suggest that this ongoing multisensory integration interacts with an internal, normative body model that is biased towards integration of inputs, in a way favoring a two-handed rather than a supernumerary representation of the bodily self (Tsakiris et al, 2010; Tsakiris 2011). In the context of our experiments, this dynamic process acts to create a coherent representation whereby the feeling that a hand that is attached to one’s own body is experienced as having been disowned, in effect replaced by the rubber hand.

But does the multisensory integration view help explain why OT correlates with the strength of the disownership experience? We speculate that because visual and tactile stimuli are usually processed within 100 ms (Vroomen & Keetels, 2010), while proprioception exceeds 200 ms (Fuentes, Gomi, & Haggard, 2012), it might be that a coherent representation focused on the rubber hand can be formed rather quickly, one that draws principally upon just the visual and tactile inputs. Delay of the proprioceptive signals might facilitate visual capture, because vision need only “capture” the tactile inputs (Pavani, Spence, & Driver, 2000). Indeed, perhaps it is for this same reason that the conscious experience of ownership seems to be the cause of drift, rather than the other way around (Abdulkarim & Ehrsson, 2016).

Some support for this view can perhaps be found from patients with schizophrenia spectrum disorders, given that, among other things, they suffer from abnormalities of proprioceptive processing (Arnfred, Raball, Morup, & Parnas, 2015). These patients also tend to exhibit notably early onset for the RHI (Peled et al., 2000; Peled, Pressman, Geva, & Modai, 2003). Here, too, it might be that proprioceptive dysfunction helps facilitate visual capture. Our point is not that early onset of the RHI is an indication of pathology, for there are participants who are especially susceptible to the RHI and begin to experience the illusion as early as 6 s after the start of synchronous stroking without any indication of pathology (Lloyd, 2007). Instead, our suggestion is that early onset of ownership and disownership, as well as the robustness of these illusions, might be partially due to normal ranges of variation in visual, tactile, and proprioceptive processing that affect how multisensory integration is achieved in any individual case.

### Why are disownership experiences less vivid than ownership experiences?

One might remain concerned that disownership for the biological hand is consistently less robust than is ownership for the rubber hand. This fact could be taken as justification for the doubt that disownership experiences occur during the standard RHI. That disownership is reported to be less vivid or less obvious than ownership is, however, not surprising, because in most experiments attention is directed primarily at the rubber hand. It necessarily follows that participant reports will reflect this intensity difference. Even in our second experiment, wherein participants were asked to monitor for disownership, a preponderance of attentional resources remained focused on the rubber hand because they had to visually attend to it. The results from our study most directly relevant to assessing whether disownership experiences occur are: (1) the significant differences obtained between synchronous and asynchronous stroking; (2) that ownership and disownership appear to be intrinsically related, at least in the sense that the more intensely participants experienced ownership for the rubber hand, the more intense were their experiences of disownership for their biological hands; and (3) that disownership was not only reflected by questionnaire response, it was also reflected by OT and ID. That is, participants proved quite capable of identifying, while undergoing the experience, when the disownership illusion began, and they did so in such a way that was consistent with their after-the-fact questionnaire responses.

## Conclusions

Contrary to the conclusions reached by Schutz-Bosbach et al. (2009), Folegatti et al. (2009), and de Vignemont (2011), our results suggest that in standard versions of the RHI, at least when the experiment is focused on the left hand, disownership experiences for the biological hand can occur. When standard RHI induction procedures are applied to the left hand, if the rubber hand is experienced as owned, it tends to follow that the biological hand will be experienced as alien. Even when care is taken to ensure that participants report not what is judged to be the case but what is experienced, they have proven quite capable of reporting the start, the end, and the duration of disownership experiences. Indeed, multiple follow-up questionnaire responses are consistent with these online reports.

## Appendix

**Table 1:** Questionnaire used in Experiments 1, 2, and 3

Adapted from Longo et al. (2008)	
Ownership	1. It seemed like I was looking directly at my own hand, rather than at a rubber hand.
	2. It seemed like the rubber hand began to resemble my real hand.
	3. It seemed like the rubber hand belonged to me.
	4. It seemed like the rubber hand was my hand.
	5. It seemed like the rubber hand was part of my body.
Disownership	6. It seemed like I was unable to move my hand.
	7. It seemed like I could have moved my hand if I had wanted.
	8. It seemed like I couldn’t really tell where my hand was.
	9. It seemed like my hand was out of my control.
	10. It seemed like my hand was moving towards the rubber hand.

**Table 2:** Questionnaire used in Experiment 3

Preston (2013)	
Ownership	1. It seemed as though I were feeling the touch in the location where I saw the fake hand being touched.
	2. It seemed as though the fake hand belonged to me.
	3. It seemed as though the fake hand was part of my body.
	4. It seemed as though the touch I felt was caused by the touch on the fake hand.
Control	5. It appeared (visually) as if the fake hand were drifting towards my own (real) hand.
	6. It seemed as if I might have more than one left hand or arm.
Disownership	7. I felt as if my real hand no longer belonged to me.
	8. It felt as though my real hand was no longer part of my body.
	9. It felt as though the fake hand replaced my own left hand.

## Footnotes

1 The authors are grateful to an anonymous reviewer who emphasized this concern about the replicability of these findings.
